# TLsub: A transfer learning based enhancement to accurately detect mutations with wide-spectrum sub-clonal proportion

**DOI:** 10.3389/fgene.2022.981269

**Published:** 2022-11-22

**Authors:** Tian Zheng

**Affiliations:** ^1^ Department of Computer Science and Technology, School of Electronic and Information Engineering, Xi’an Jiaotong University, Xi’an, China; ^2^ Institute of Data Science and Information Quality, Shaanxi Engineering Research Center of Medical and Health Big Data, Xi’an Jiaotong University, Xi’an, China

**Keywords:** genetics, structural variation, machine learning, next generation sequencing, mutation detection

## Abstract

Mutation detecting is a routine work for sequencing data analysis and the trading of existing tools often involves the combinations of signals on a set of overlapped sequencing reads. However, the subclonal mutations, which are reported to contribute to tumor recurrence and metastasis, are sometimes eliminated by existing signals. When the clonal proportion decreases, signals often present ambiguous, while complicated interactions among signals break the IID assumption for most of the machine learning models. Although the mutation callers could lower the thresholds, false positives are significantly introduced. The main aim here was to detect the subclonal mutations with high specificity from the scenario of ambiguous sample purities or clonal proportions. We proposed a novel machine learning approach for filtering false positive calls to accurately detect mutations with wide spectrum subclonal proportion. We have carried out a series of experiments on both simulated and real datasets, and compared to several state-of-art approaches, including freebayes, MuTect2, Sentieon and SiNVICT. The results demonstrated that the proposed method adapts well to different diluted sequencing signals and can significantly reduce the false positive when detecting subclonal mutations. The codes have been uploaded at https://github.com/TrinaZ/TL-fpFilter for academic usage only.

## 1 Introduction

Mutations may alter the reading frame of protein coding sequences and have been strongly implicated in neurodevelopmental disorders, cardiovascular diseases, cancers and many other human diseases (Fang et al., 2016). Mutation calling *via* genomic sequencing, also named variant detection, has become a routine task in cancer diagnosis and precision treatments. The existing mature methods depend essentially on combinations of statistical signals (also known as features in machine learning) on a set of mutation-centred overlapped reads for detection and filtering (Cibulskis et al., 2013; Tian et al., 2020). One frequent concern in cancer genomics is that tumor samples are always heterogeneous, composed of tumor cells, stromal contamination and normal cells (Tang et al., 2016). Since one goal of a somatic pipeline is to establish the catalog of the somatic mutations occurring in the tumor cells, at this time, the tumor somatic mutations are subclones, it is important to take into consideration the composition of the sample. The proportion of tumor cells in total cells is usually summarized as “tumor purity” (which approximately equals to the proportion of tumor cell somatic mutations in total sample somatic mutations and is usually summarized as “clonal proportion”) (Arora et al., 2019). For most cancer types in The Cancer Genome Atlas (TCGA), the content of normal cells in tumor samples is generally between 30% and 70%. Previous studies have shown that tumor purity will have an important impact on the gene data in tumor research. When detecting subclonal mutations, existing signals are often diluted due to insufficient abundance and may lead to deviation in biological interpretation results (Stratford et al., 2016; Rhee et al., 2018; Koudijs et al., 2019).

**FIGURE 1:**
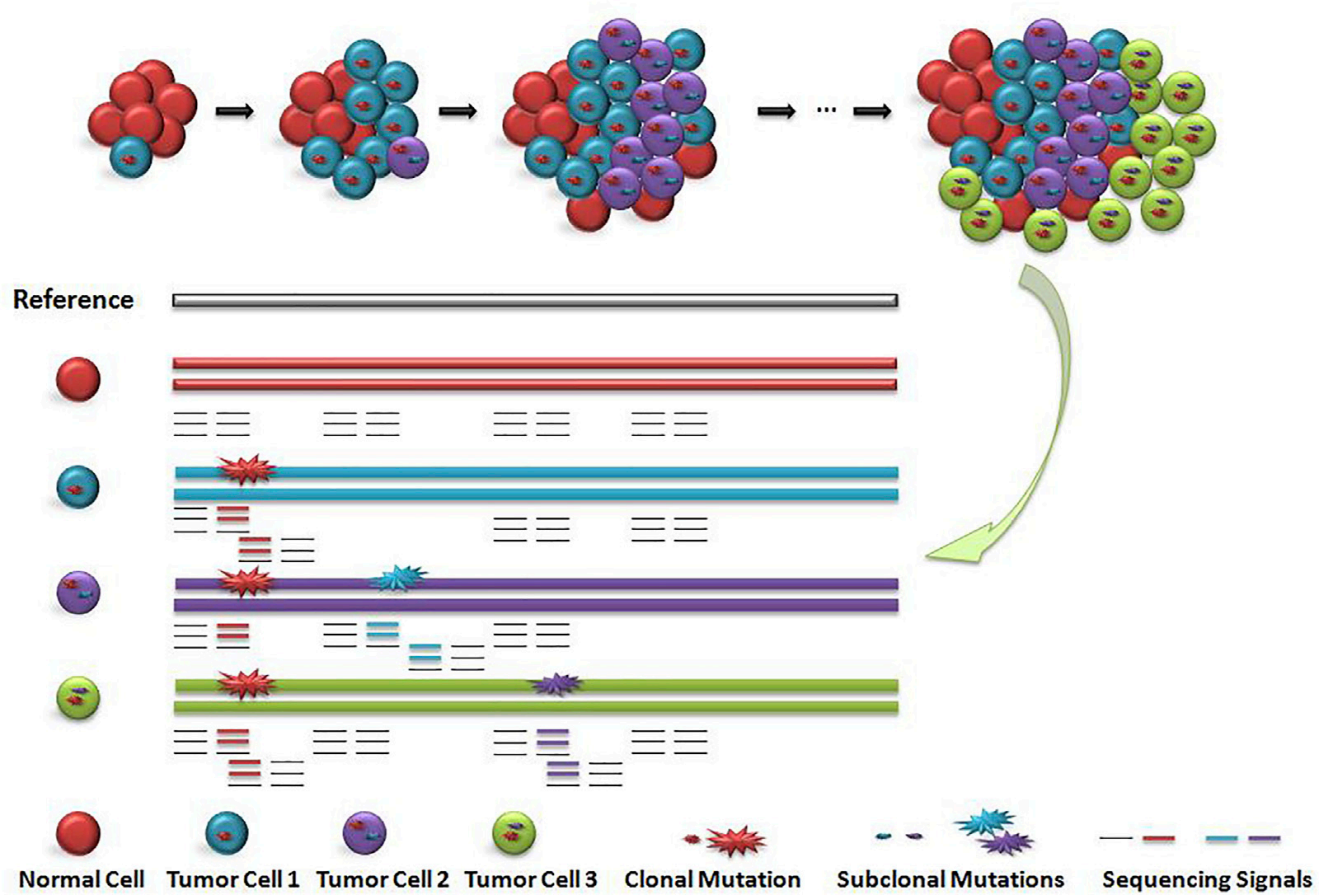
Schematic diagram of subclonal variation and detection signals. When the main clone and sub clone were included, the cell distribution and sequencing signal were diluted. Red circle indicates the main clone variation, blue circle and purple circle indicate the sequencing signal of subclone variation. The value of sequencing signal of sub-clone variation is closely related to the number and proportion of corresponding cells, but the distribution of the number of cells including main clone and subclone is unknown, so the value of sequencing signal is difficult to determine.

Existing detection signals for short read data are mainly divided into five categories (Ho et al., 2020): 1) read depth (Abyzov et al., 2011; Klambauer et al., 2012), 2) paired-end read (Chen et al., 2009), 3) split read (Ye et al., 2009), (iv)*de novo* assembly (H, L et al., 2015; Chen et al., 2014) and their combinition (Rausch et al., 2012; Layer et al., 2014; Chen et al., 2016). The ensemble strategy by using multiple discrete approaches to detect variations and then integrating all variant call sets to generate a unified call set is widely adopted by large-scale human genome studies (Nagasaki et al., 2015; Sudmant et al., 2015). It is a big challenge to filter out false positive variant calls. As shown in Figure 1, cells with different colors have subclonal variation with different proportions. How to accurately identify the subclonal variation of tumor tissue is a computational problem to be solved.

A novel research varied tumor purity and coverage through biological experiments to detect the actual accuracy change of mutation callers when the subclonal mutation signal was diluted, and the results showed that when the tumor purity is less than 50%, the F-score of all callers dropped precipitously, generally less than 20%. When tumor purity was 20% or lower, the accuracy and precision of the mutation callers are significantly decreased (Xiao et al., 2021). 166 false positives can be introduced per mega-base by every 2% reduction in the proportion of tumor purity (Cibulskis et al., 2013) and the precision of mutation drops rapidly (even less than 25%) when the clonal proportion is less than 50% (Koudijs et al., 2019).

We tried to explain this phenomenon from two aspects, combined with our understanding of the existing mutation detection softwares and false positive filtering algorithms. First, the existing methods mainly set thresholds and perform filtering based on the value difference of a series of signals between the real mutations and the false positives (Cibulskis et al., 2013; Viola et al., 2018). The basis of threshold setting is the probability of signal distribution. According to the probability density function, whether it is artificial design or machine learning, the inflection point of filtering can be found. All signal values may be affected by the proportion of subclonal mutations, but the formula for impact is hard to quantify in three-dimensional space, and the probability density function is essentially different for different signals. Regardless of how the signal threshold setting is changed, it only partially reduces false positives and cannot fundamentally filter false positives and accurately detect subclonal mutations, not to mention the wide-spectrum range of clonal proportions. Second, the clonal proportion is a continuous variable, and no training set can enumerate all possible values. The existing machine learning methods train the model under fixed clonal proportions, but this leads to poor application of the model on other datasets with different clonal proportions (Garrison et al., 2012; Can et al., 2016; Freed et al., 2017). A TCGA-related research showed that many subclonal variations are still missed after 41.8% false positives are filtered out on low-quality samples even used different variation detection and multiple filter software, and the average detection rate is only 72.5% (Gao et al., 2019). These challenges hurt the specificities of the existing approaches when applied to cancer sequencing data. It is not practical to train the corresponding model for each diluted signal. We need to deconstruct the relationship between the signals and the clonal proportion and to explore the binary separable relationship to accurately identify the subclonal mutations.

Motivated by these, we focused on the accurate detection of subclonal mutations and filtering of false positive mutation calls. We proposed a novel approach to the scenario of various clonal proportions that overcomes these limitations by means of a transfer learning technique. On the basis of observing the relationship between the sequencing signal and the proportion of the subclonal mutations, we reconstructed a new regenerative Hilbert space and mapped the sequencing signal to it, making the false positive and the true mutation binary separable in the new high-dimensional space. We carried out a series of experiments on both simulated and real datasets. The results were compared to state-of-art approaches, including MuTect2 (Cibulskis et al., 2013), Freebayes (Garrison et al., 2012), SiNVICT (Can et al., 2016), and Sentieon (Freed et al., 2017). The results demonstrated that the proposed method adapts well to different datasets with wide-spectrum clonal proportions and can significantly reduce false positives. The code has been uploaded at https://github.com/TrinaZ/TL-fpFilter for academic use only.

## 2 Results

**FIGURE 2:**
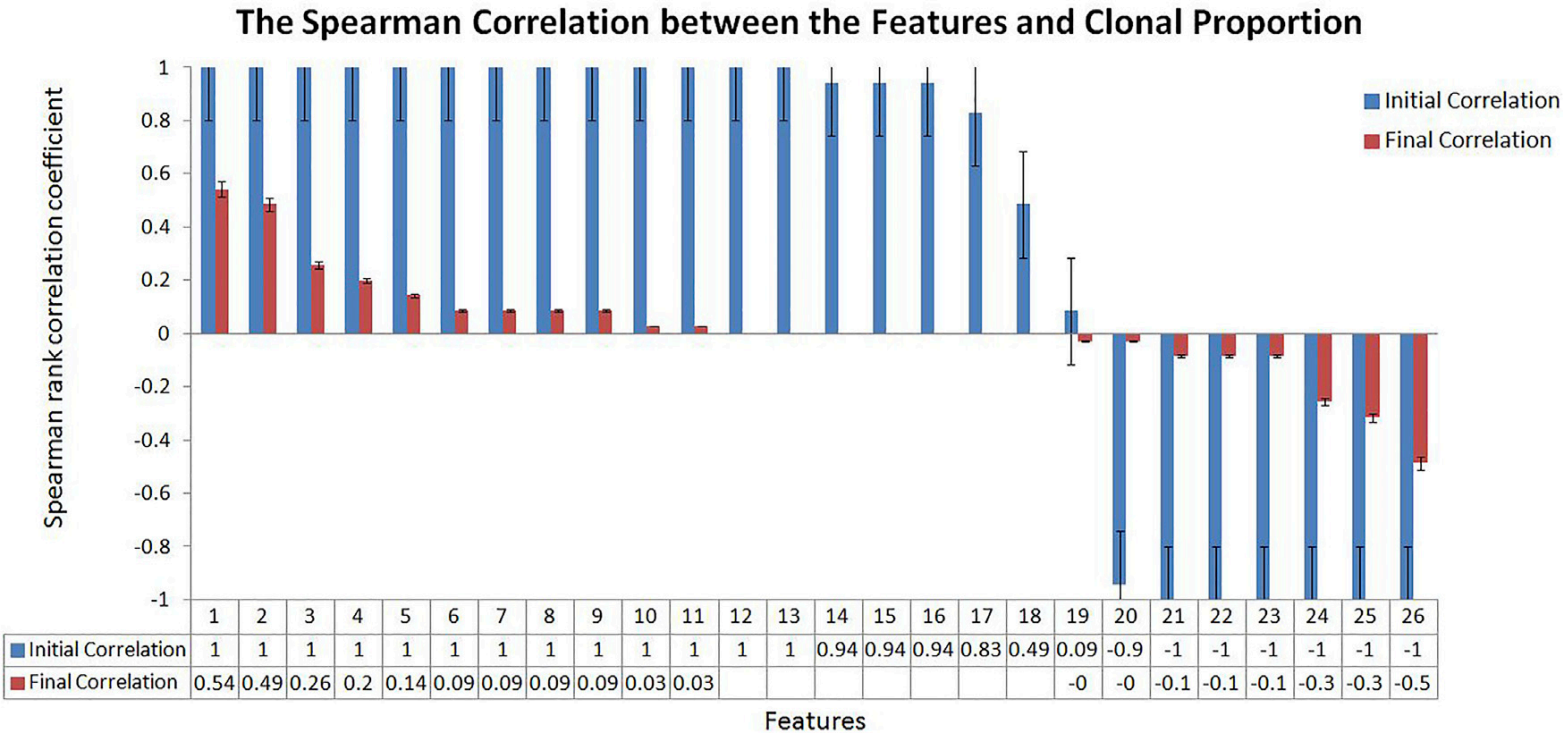
The Spearman Correlation between the Features and Clonal Proportion. According to the Spearman correlation coefficient of sequencing signal and the diluted sequencing signal, it can be seen that most of the signals have strong correlation with the clonal proportion (Initial Correlation in Blue). The results showed that the correlation coefficient between the transferred features and sample purity was significantly reduced (Final Correlation in Red).

We tried to weaken the interference of sample abundance such as tumor purity and clonal proportion on the variation detection signals by algorithm design. To measure the effect of the proposed algorithm, we calculated the correlation between the variation detection features and the clonal proportion as shown in Figure 2. We extracted a 100 Mbps reference sequence from human genome 19 (hg19) and obtained the value of sequencing signal data under different clonal proportions by simulation software. We simulated the clonal proportion every 5% points from 0 to 1 and obtained the value of signals at different proportion. 26 popular features are extracted from the Variant Call Format file and their descriptions with calculated formula are shown in Section 3.1; Table 1. We calculated the Spearman rank correlation coefficient between each feature and clonal proportion. As we known, the closer the absolute value of the correlation coefficient is to 1, the stronger the correlation between the two factors is, and the closer it is to 0, the weaker the correlation is. The blue results in Figure 2 indicate the relationship between features and clonal proportion before algorithm processing. The results in red indicate the correlation after algorithm processing. The black error line on the histogram shows the possible error related to each data mark in the data series in graphical form. In this figure, it is set as 5%, and it is allowed to have a possible error of plus or minus 5%.

**TABLE 1:** Features list.

Feature	Definition	Extraction source
IMPRECISE	Imprecise structural variation	IMPRECISE value in INFO column
CIPOS	Confidence interval around POS for imprecise variants	CIPOS value in INFO column
CIEND	Confidence interval around END for imprecise variants	CIEND value in INFO column
CIPOS95	Confidence interval (95%) around POS for imprecise variants	CIPOS95 value in INFO column
CIEND95	Confidence interval (95%) around END for imprecise variants	CIEND95 value in INFO column
GT	Genotype	GT value in FORMAT column
SU	Number of pieces of evidence supporting the variant	SU value in FORMAT column
PE	Number of paired-end reads supporting the variant	PE value in INFO column
SR	Number of split reads supporting the variant	SR value in INFO column
GQ	Genotype quality	GQ value in FORMAT column
SQ	Phred-scaled probability that this site is variant (non-reference in this sample)	QUAL column
GL1	Genotype Likelihood, log10-scaled likelihoods of the data given the called genotype for each possible genotype generated from the references and alternate alleles given the sample ploidy	First GL value in FORMAT column
GL2		Second GL value in FORMAT column
GL3		Third GL value in FORMAT column
DP	Read depth	DP value in INFO column
RO	References allele observation count, with partial observations recorded fractionally	RO value in INFO column
AO	Alternate allele observations, with partial observations recorded fractionally	AO value in INFO column
QR	Sum of quality of references observations	RO value in FORMAT column
QA	Sum of quality of alternate observations	AO value in FORMAT column
RS	References allele split-read observation count, with partial observations recorded fractionally	RS value in FORMAT column
AS	Alternate allele split-read observation count, with partial observations recorded fractionally	AS value in FORMAT column
ASC	Alternate allele clipped-read observation count, with partial observations recorded fractionally	ASC value in FORMAT column
RP	References allele paired-end observation count, with partial observations recorded fractionally	RP value in FORMAT column
AP	Alternate allele paired-end observation count, with partial observations recorded fractionally	AP value in FORMAT column
AB	Allele balance, fraction of observations from alternate allele, QA/(QR + QA)	QA/(QR + QA)
CN	Copy number of structural variant segment	CN value in FORMAT column

The results in blue (initial correlation) demonstrate that almost all the signals are strongly correlated with the clonal proportion (±1). The results in red (final correlation) demonstrated that the correlation was significantly reduced (±0.5). We conducted a detailed analysis on the features that were not strongly correlated with the clonal proportion, PE and AS, defined as the number of paired-end reads supporting the variant and the alternate allele split-read observation count, with partial observations recorded fractionally. The Spearman correlation coefficients between them and the clonal proportion are 0.49 and 0.09, respectively. The reason may be that the correlation between each pair of signals and the clonal proportion is not high. PE values are generally large, and the influence of the proportion is limited. As the values of AS are generally small, the influence is still low.

**FIGURE 3:**
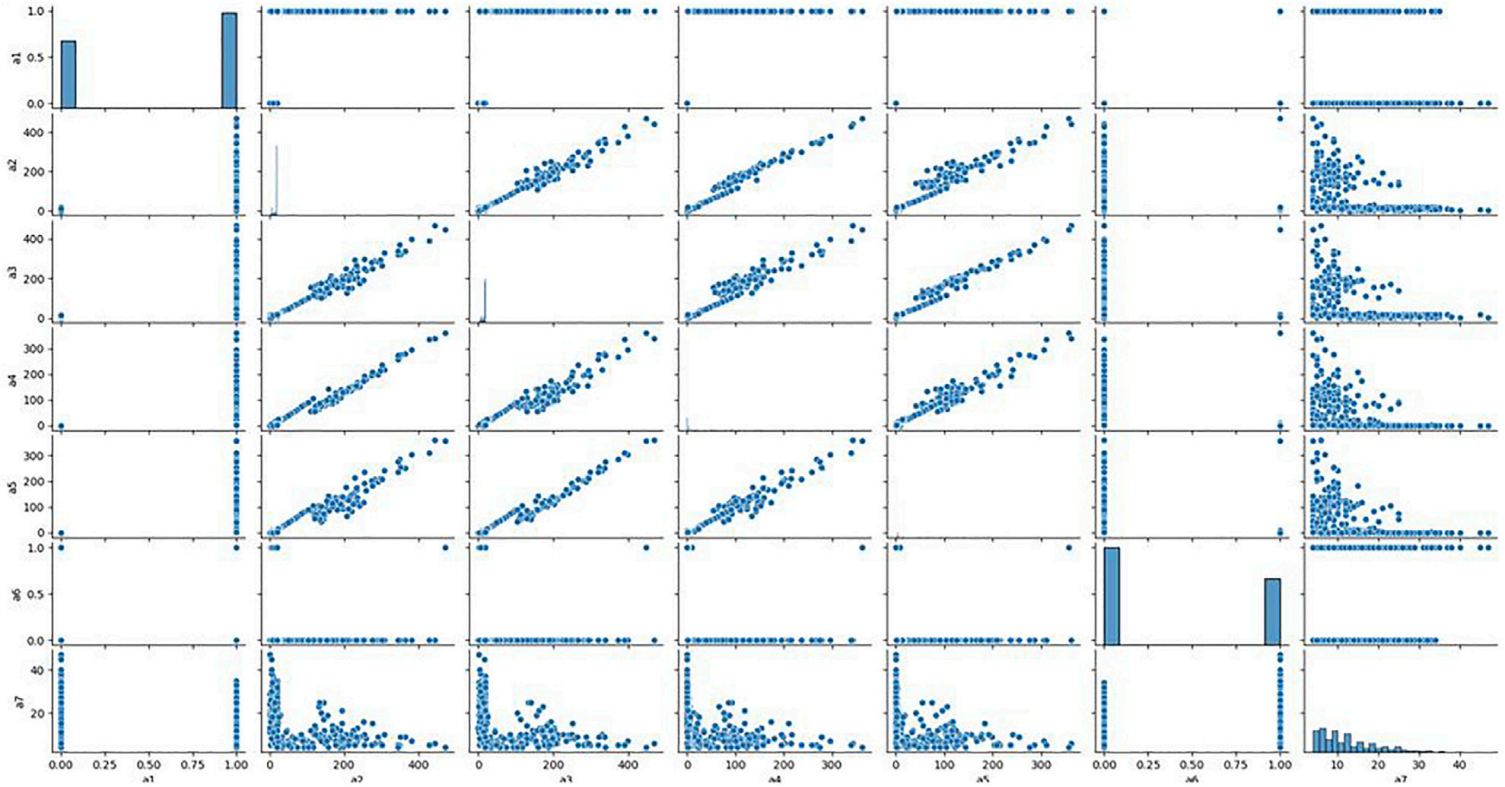
Relationship between the most relevant features. We analyzed the correlation between the seven features most related to the clone proportion in the cross clone proportion correlation analysis, and found that there was no consistent correlation between the seven features from a two-dimensional perspective.

To further quantitatively analyse the specific relationship between each signal and the clonal proportion, we visualized the correlation matrix of the six signals, CIEND, SQ, RP, CN, RO, and CIPOS, between different clonal proportions (Supplementary Figure S1 in Supplementary Material). The heatmap figures demonstrated that the correlation between each signal and clonal proportion is different. Complicated interactions occur among the signals and break the independent co-distribution assumption of classic learning models. This further illustrates that the use of ordinary machine learning methods cannot linearly strip the impact of clonal proportion on different signals. The baseline obtained by training signals under a certain clonal proportion is effective under the corresponding determination of clonal proportion, but the correlation between signals and proportion has changed in other values, and the classification baseline is no longer applicable. It is unrealistic to retrain under continuously changing proportion conditions. We pick out the seven most relevant features and draw the correlation diagram as shown in Figure 3 to show their similarity. The results show that the features with strong correlation can not completely replace each other, and the influence of sample abundance should not be eliminated by deleting features.

### 2.1 Performance on simulated data

To verify the performance of the proposed method under clear evaluation benchmarks, we conducted tests on simulated datasets. We sampled a 1 M base pair (bps) region from the human reference genome and randomly planted 200 structural variations in each data set, including insertion, deletion, complex indel, copy number variant (CNV), and their combination. We set the length of variation to 800–1,200 bps and the length of CNV to 1,000 bps and set both to follow a normal distribution. The variation interval was larger than 2000 bps. We set an elevated region to 1,000 bps longer than its own length and set the mutation rate of the region to 0.01. Some associated SNVs (single nucleotide variants) were planted in the preset elevated region, and the background mutation rate was set to 0.0001. Approximately one-fourth of the inserted fragments of the complex indel came from nearby regions. Twenty-five percent of CNVs were accompanied by a deletion, and 15% of CNVs were accompanied by an insertion. The length of the sequencing reads was set to 100 bps, the distribution of insert sizes was set to follow a normal distribution with a 500 bps mean and 15 bps standard deviation, and the sequence error rate of read sampling was considered to be 0.001. The structure variation was detected by SpeedSeq (Chiang et al., 2015) with the default parameters, and the signals were extracted from the output VCF(Variant Call Format) file. The true and false positive labels were obtained by comparison with the original insertion file.

We repeated the above process 20 times and obtained a data set containing 4,000 samples. Furthermore, we used the classic method of data sampling to balance the positive and negative categories: undersampling a large number of categories (classic easyensemble) and oversampling a small number of categories (classic SMOTE). Finally, for each clonal proportion simulated dataset, the positive and negative categories were balanced. We collected 4,000 samples each for clonal proportions of 5%, 10%, 15%, 20%, 25%, and 30% and used them as the source domain and target domain. The confusion matrix is a standard format for precision evaluation, in which TN means true negative, FP means false positive, FN means false negative, and TP means true positive. The elements on the main diagonal of the confusion matrix correspond to the correct classification, while the other elements tell us how many samples in a category are incorrectly classified into other categories. The accuracy results are shown in Table 2, and the exact precision, recall and F1-scores are given in Supplementary Tables S1-S3. Each of them is the average of five repeated experiments.

**TABLE 2:** Benchmark results of the method on simulated data.

TSP SSP	Accuracy (%)					
	5%	10%	15%	20%	25%	30%
5%		80.18	81.10	80.43	72.75	72.83
10%	84.83		89.03	89.23	85.05	82.45
15%	83.30	91.45		90.53	90.55	89.95
20%	79.38	86.95	86.18		91.03	91.98
25%	73.90	83.00	87.85	91.75		91.78
30%	73.93	81.63	80.48	91.10	89.50	
Average	79.07	84.64	84.93	88.61	85.78	85.80
Output	**81.18**	**87.70**	**89.93**	**91.95**	**91.30**	**92.10**

The best results are highlighted in boldface.

The results showed that the accuracy of false positive filtering is stable above 80%, and the average of the six results is 89.03%, which demonstrated that the proposed method can successfully filter out false positives from the structural variation detection. The accuracy of the final output voting result is stable and higher than average, which also proves the necessity of applying the voting algorithm. Moreover, we found that the effect of the transfer from high to low propagation is higher than the accuracy of transfer from low to high clonal proportions, which is consistent with the conclusion that false positives increase as the detected preset clonal proportion decreases.

#### 2.1.1 Method comparison on mixed-proportion simulated datasets

In real situations, many different subclonal mutations with different clonal proportions may exist at the same time. To sufficiently demonstrate the performance of their framework on real data and further simulate the real cancer detection situation, we mixed different proportions of simulated data to give a better estimate of the performance of the proposed framework. We set the clonal propagation to follow a normal distribution, with an average value of 70% and a variance of 10%. The number of mutations was randomly generated in the interval [2200, 2400]. The parameters are set as follows: error rate, 0.01; min-depth, 100; left-strand-bias, 0.3; right-strand-bias, 0.7; read-end-fraction, 0.01; qscore-cut-off to 20, use-poisson-germline specified with value to 1, disable-lvl to 5, filter specified with value to 0. Each experiment is the average of 5 independent replicates. In addition, we compared the proposed method to a set of popular variation detection tools that performed outstandingly in detecting low-proportion sample variation, including Freebayes (Garrison et al., 2012), SiNVICT (Can et al., 2016), Sentieon-2019 (Freed et al., 2017) and GATK3.8 MuTect2 (Cibulskis et al., 2013) (Table 3).

**TABLE 3:** Comparison results on mixed purity samples.

SP(%)	Precision (%)				
	*TL-fp Filter*	*freebayes*	*Sentieon*	*SiNVICT*	*MuTect2*
R1	87.02	53.19	**87.13**	78.59	79.00
R2	**88.63**	53.41	88.17	79.19	80.03
R3	86.24	53.69	**87.65**	79.63	79.73
R4	**91.32**	53.70	88.52	79.71	79.52
R5	**89.98**	53.44	87.62	78.97	80.89
5	**89.23**	33.88	87.49	74.81	77.37
10	**89.50**	53.35	87.71	78.98	79.62
15	**91.02**	53.47	87.57	81.00	79.08
20	**90.82**	53.02	86.50	76.69	78.35
25	**90.32**	53.87	87.98	79.93	79.85
30	**90.50**	53.47	87.90	79.08	78.90

The best results are highlighted in boldface. SP represents the sample purity. R1-5 represent five mixed sample experiments. 5–30 represent fixed purity sample experiments under the same conditions.

R1-5 represent five mixed sample experiments, and 5–30 represent fixed clonal proportion sample experiments under the same conditions. The results demonstrated that the proposed method adapts well to the mixed proportion datasets. Among the four comparison methods, Sentieon is least affected by clonal proportion, with a false positive rate of approximately 13%. The false positive rates of other methods are higher than 20%, and Freebayes was most affected by the clonal proportion, with a false positive rate higher than 40%. The proposed method can significantly filter false positives, and the highest false positive rate can be reduced by 55.35%. We further analysed the results and found that the general performance of each method on the fixed proportion dataset is slightly better than that on the mixed dataset. This is because the average value of the mixed dataset is 70%, and the fixed variance is less than 30%. This is also consistent with the influence of clonal proportion on the detection accuracy. Among them, the proposed method performs outstandingly in low-clonal-proportion fixed datasets and mixed datasets.

#### 2.1.2 Quantifying the effect of the proposed stage I

As proof of the value of the proposed method, we quantified the effect of transfer learning for false positive filtering. The results are shown in Table 4, which compares the performance of the model trained only on the target dataset (baseline, listed in column “B”) with the model transferred from the source dataset to the target dataset (listed in column “TCA”). For the comparison baseline samples, the model is applied with the autofit parameters and without TCA. For the TCA samples, the model exactly follows the proposed stages. Each result is the average of five repeated experiments. The results demonstrated that the transfer component analysis improved the average accuracy, precision, recall and F1-scores over the baseline. More specifically, TCA led to average improvements of 22.18% in accuracy, 7.92% in precision, 12.89% in F1-score, and 6.11% in recall rate. Upon further analysis, TCA played a greater role in samples with low clonal proportions.

**TABLE 4:** Performance values in terms of accuracy, recall, precision and F1-score for Baseline (B) and Transfer Component Analysis (TCA).

SP (%)	Accuracy (%)		Recall (%)		Precision (%)		F1-score (%)	
	B	TCA	B	TCA	B	TCA	B	TCA
5	62.90	**81.18**	58.85	**72.80**	81.90	**87.45**	68.48	**79.45**
10	69.60	**87.70**	77.80	**84.20**	75.49	**89.53**	76.62	**86.78**
15	74.45	**89.73**	89.40	**93.00**	85.20	**86.92**	87.25	**89.86**
20	60.30	**91.95**	89.10	**92.25**	76.41	**91.70**	82.27	**91.97**
25	64.27	**91.30**	88.60	**90.70**	84.36	**91.80**	86.43	**91.25**
30	69.37	**92.10**	85.05	**92.50**	88.32	**91.77**	86.65	**92.13**

The best results are highlighted in boldface.

#### 2.1.3 Quantifying the sensitivity to parameters

We conducted parameter adjustment experiments and quantified the sensitivity of the method to the following parameters, which include the dimensionalities of the latent spaces of TCA, the largest number of decision trees (n_estimators), the maximum number of features considered in the split (max_features), the maximum depth of the decision tree (max_depth), and the minimum number of samples required for the internal node subdivision (min_samples_split).

##### 2.1.3.1 The dimensionalities of the latent spaces

We first tested how the dimensionalities of the latent spaces in TCA affect the classification performance. We used linear kernels and set the dimension of the latent space to vary from 5 to 23. During the experiment, we found that when the value of the dimensionalities of the latent spaces was 24–26, there was no correct result because the total number of features was 26. The exact results are shown in Table 5. When the value is in the range of [10, 23], the accuracy of the method remains high, but when the value is 5, there is no output due to the low dimensionality. Moreover, note that within the allowable range, the higher the dimension of the latent space is, the higher the accuracy of the method, the higher the cost, the larger the calculation amount and the longer the training time. Therefore, in subsequent experiments, we fixed the value of this parameter to 15 in a trade-off between accuracy and computational complexity.

**TABLE 5:** The test results of the dimensionalities of the latent space.

Sample purity (%)	The dimension of the latent space				
	5	10	15	20	23
5	*	79.43	79.43	80.05	**80.10**
10	*	85.43	86.63	87.13	**87.70**
15	*	88.73	89.15	88.90	**89.73**
20	*	90.98	90.50	90.75	**90.93**
25	*	89.13	89.85	89.75	**89.98**
30	*	91.05	89.88	89.70	**90.33**

The best results are highlighted in boldface.

##### 2.1.3.2 n_estimators

The value of n_estimators represents the maximum number of decision trees of in the extra tree classifier, the maximum number of iterations of weak learners, or the maximum number of weak learners. The extra tree classifier has relatively few important parameters, and the main concern is n_estimators. Generally, if the value of n_estimators is too small, the extra tree classifier is easily underfitted; if the value of n_estimators is too large, the calculation amount will be too large. When the value of n_estimators reaches a certain number, the model improvement obtained by increasing n_estimators will be very small, so a moderate value is generally selected. The default is 100, and in this case, the best value of n_estimators based on the experimental results is 60–70. The results are listed in Supplementary Table S4.

##### 2.1.3.3 max_features

The max_features represents the maximum number of features considered in the extra tree classifier. In this problem, the number of features is 26, and the default max_features is “auto,” which means that the maximum number of features considered is $21\left(n-\sqrt{n}\right)$ . In general, we can use the default “auto.” We tested the values from 0 to 23 and found that it reached the optimal value when the maximum number of features was 19. The results are listed in Supplementary Table S5.

##### 2.1.3.4 max_depth

The max_depth indicates the maximum depth of the decision tree in the extra tree classifier, which can be left blank by default. If it is left blank, the decision tree will not limit the depth of the subtree when it is built. In general, this value can be ignored when there are few data or features. If the number of model samples and features is high, it is recommended to limit the max_depth. The specific value depends on the distribution of the data, and the common recommended range is [10, 100]. We debugged in the variable range of this parameter and found that the experimental results are not sensitive to this parameter. The results are listed in Supplementary Table S6, and the optimal value of max_depth in this case can be 7–11.

##### 2.1.3.5 min_samples_split

The value of min_samples_split represents the minimum number of samples required for internal node subdivision, which can limit the ability of the sub tree to subdivide. If the number of samples of a node is less than the value of min_samples_split, it will stop selecting the best partition feature. The default value of the min_samples_split is 2. See Supplementary Table S7 for the experimental results of adjusting this parameter. The results demonstrated that this parameter has little effect on these data, and the proposed method has the highest performance when the value of min_samples_split is set to 1.

##### 2.1.3.6 min_samples_leaf

The min_samples_leaf represents the minimum number of samples required for each leaf node (Supplementary Table S8). We debugged this parameter for a comprehensive experiment, and the results showed the best effect when this parameter is 1.

#### 2.1.4 Quantifying the boundary of the proposed method

To further test the application boundary of the proposed method, we varied the number of available samples and the range of the label error rate and recorded the method performance.

1) The minimum applicable number of samples.

Generally, in the application of machine learning algorithms, the smaller the number of samples is, the lower the accuracy of the algorithm will be. We selected three test datasets, which included 200, 400, and 600 samples, to test the method performance on small samples. The ratio of positive and negative labels was set to 1:1 to eliminate the possible impact of data imbalance. The values of each parameter were set to the optimal values of the above experiments. The results are shown in Supplementary Table S9, which demonstrated that our method is still effective when the number of samples is less than 1,000, and the effect is not significantly different.

2) The maximum allowable range of label error rate.

In addition to the applicable sample size, the sample error rate may affect the applicability of the software. To further test the application boundary of our method, we varied the label error rate and recorded the performance of the proposed method. The label error rate refers to the frequency of the error in which a positive sample is marked as negative or a negative sample is marked as positive. We set the sample size to 4,000 and the value of each parameter to the optimal values of the above experiments. The results are shown in Supplementary Table S10. We can see that our method performs well in all aspects of the indicators as long as the error rate is less than 30%, and the results are significantly stable. However, it should be noted that when the clonal proportion is 5% and the label error rate is 20% or 30%, the recall rate is relatively low.

### 2.2 Performance on real data

To further test the performance of this method, we carried out experiments on real data sets. We selected 12 groups of panel data and 12 sets of whole-exome sequencing (WES) data from the real database and carried out experiments. It should be noted that, due to the importance and scarcity of real human gene data, we use 12 groups of data here, which should be highlighted as a limitation.

#### 2.2.1 The experiments on the panel datasets

We obtained six groups of lung cancer data and six groups of breast cancer data from the public database to test the performance of the proposed method on real data (Ma et al., 2017). Due to the characteristics of the formation principle of cancer species, the clonal proportions of these two types of cancer can be extremely low, and their detection accuracy is seriously affected by tumor purity. All clinical information was removed, patients were numbered by a random target, and all germline mutations were also removed before we obtained the data. The raw data have already been processed on the public database, following the pipeline in which the raw sequence read was mapped by BWA-0.7.5, and GATK3.8 MuTect2 and CNVkit were used to detect the true structural variation information. SpeedSeq was used to extract the VCF standard file, and the label was annotated by comparison with the standard results in the public database.

We chose a test sample size of 100 and randomly selected 50 true positive samples and 50 false positive samples. We set the parameter to the best results of the tuning experiment. Because it is difficult to simulate data on batch errors and population characteristics, there is a large gap between the real data and the simulated data. We randomly selected 4 of the real data sets as the source domain for training (training datasets) and made the remaining eight samples the final test sets. The results are shown in Table 6. In addition, we listed the results of GATK3.8MuTect2 on the same samples. The real data we choose is panel datasets, and its parameter settings have been adjusted to detect low-frequency mutations, which is preferable to find low-frequency mutations. There are many false positives, resulting in a very low detection effect of MuTect. Experimental results show that our method can greatly filter false positives in this situation.

**TABLE 6:** The experiment results on real datasets.

	The proposed method (%)				MuTect2			
	F-1 score	Recall	Accuracy	Precision	Candidate	Detected	Validated	Validation rate (%)
1	76.52	88.00	73.00	67.69	1968	1,501	506	33.71
2	82.57	90.00	81.00	76.27	2437	1,502	474	31.56
3	80.41	78.00	81.00	82.98	2598	1,678	491	39.06
4	87.38	90.00	87.00	84.91	2433	1,506	500	33.20
5	85.71	96.00	84.00	77.42	2752	1,616	501	31.00
6	90.38	94.00	90.00	87.04	2129	1,477	502	33.98
7	86.54	90.00	86.00	83.33	2937	1896	550	29.00
8	88.99	88.00	89.00	90.00	2848	1,539	512	33.27

#### 2.2.2 The experiments on the WES datasets

To further test the performance of the proposed method on real data, we applied it to real WES datasets. We obtained 12 groups of lung cancer data detected by the We6v chip from the public database (Table 7). All clinical information was removed, patients were numbered by a random target, and all germline mutations were removed before we obtained the data. The raw data were already processed on the public database, following a pipeline in which the raw sequence read was mapped by BWA-0.7.5, and GATK3.8MuTect2 and TNscope 2018.3 were used to detect the true structural variation information. The results of Mutect2 were filtered by FilterMutectCalls and the low_t_alt_frac; t_lod_fstar of TNscope. The sequencing depth was 300×/500×. The label was annotated by comparison with the public database and the overlapping results of several software detection results. Other experimental settings are consistent with the panel data experiment.

**TABLE 7:** The experiment results on real datasets.

	The proposed method (%)				Mutect2		
	F-1 score	Recall	Accuracy	Precision	Detected	Validated	Validation rate (%)
1	91.76	91.43	93.90	92.08	14146	9606	67.90
2	91.17	90.76	93.83	91.59	14466	9454	65.35
3	91.92	91.49	93.60	92.35	13906	8495	61.08
4	87.12	86.74	93.95	87.51	14746	8877	60.19
5	90.27	89.89	94.15	90.66	15271	9566	62.64
6	88.00	87.62	94.10	88.38	15168	9559	63.02
7	93.07	92.83	96.40	93.31	25224	15433	61.18
8	94.86	94.63	96.57	95.10	26499	16512	62.31
9	89.83	89.66	97.45	89.99	35934	19577	54.48
10	77.04	76.80	95.55	77.29	20315	13277	65.36
11	94.37	94.02	94.82	94.73	17366	9775	56.29
12	91.04	90.63	93.73	91.46	14230	9871	69.37

The results demonstrated that the proposed method has good adaptability to real data and even better adaptability to simulated data. We mixed two types of cancer as the source domain, and the results showed that the proposed method has no training differences or preferences for cancer types and can effectively overcome the impact of diluted sequencing signals on real data and filter false positives. The results showed that 1) dilution of sequencing signals is obvious in cancers with low clonal proportions, and 2) the proposed method can correct this effect and effectively reduce false positives.

## 3 Materials and methods

**FIGURE 4:**
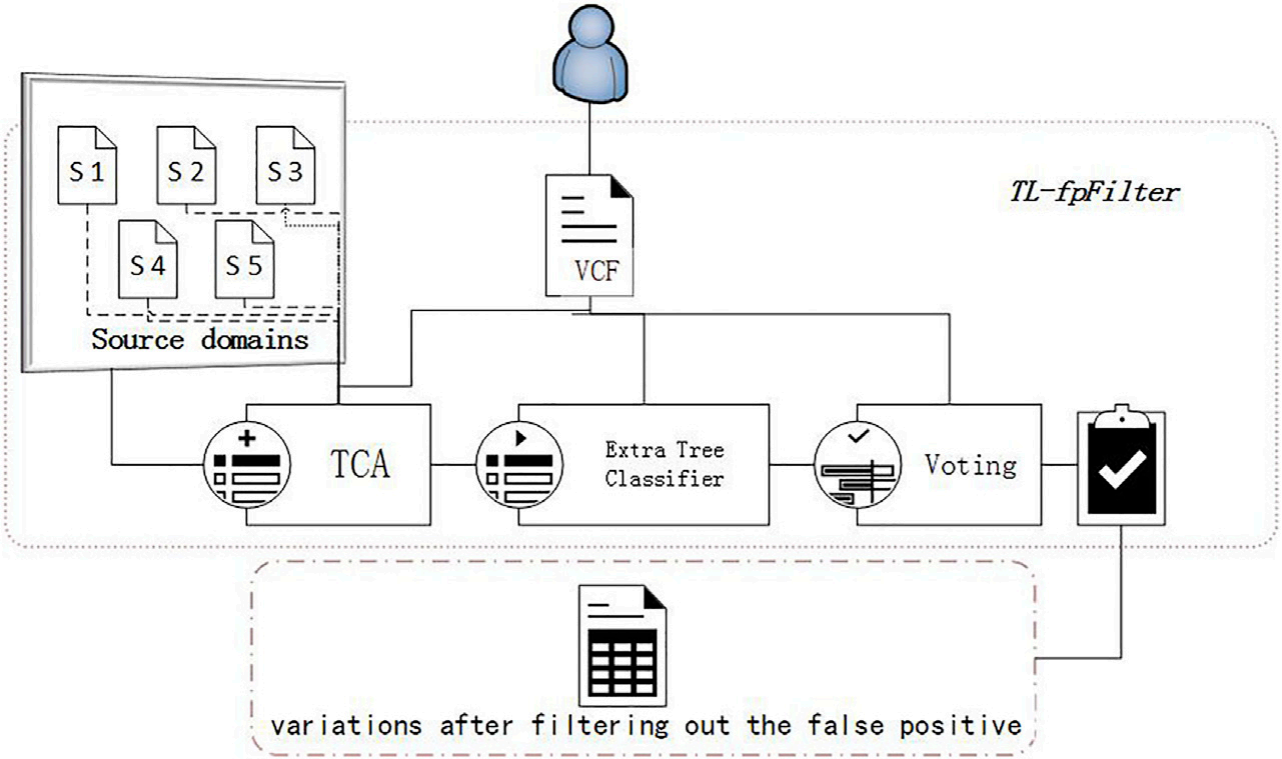
The flow diagram of the proposed method.

The overall operation schematic of the proposed method is shown in Figure 4. For users, the input of the proposed method is the standard VCF file, and the output is the set of mutations after filtering out the false positives. It should be noticed that VCF files can be non standard between different software. The VCF file format for this method is the format specified in The Variant Call Format (VCF) Version 4.1 Specification, which master version can be found at https://github.com/samtools/hts-specs. The software supports the user in inputting a series of data with different batches and training with their specific source domains. The calculation steps are detailed in stages as follows. First, the proposed approach incorporates a comprehensive set of features (signals) according to the existing strategies. Then, it requires at least two training sets with different proportions. The framework first trains the models according to one set, which is defined as a source domain. The trained models focus on the associations between the features and true mutations. Next, when the other set is input for training, the framework not only trains another source domain but also focuses on the transformations among the features between the source domains. Now, when other fixed clonal proportions are considered, the framework can generate, perhaps roughly, models for the new group of proportions according to the source domains and the transformations. The transfer learning algorithms can automatically reconstruct models applicable to other clonal proportion datasets based on knowledge of the previously trained false positive filter model.

### 3.1 Stage I: Transfer component analysis

Specifically, for each mutation call, a fixed set of predefined features is computed (Table 1) to obtain a handcrafted feature vector consisting of effective standards for mutation detection. We define the feature matrix as X , the marginal probability distribution of inputs as P(X) , $X=\left\{x_{1},x_{2},\;\ldots \;,x_{26}\right\}\in \chi$ as a learning dataset sample, and X as the space of all document vectors. Each vector corresponds to a structural variation filter result with two states: false positive and true positive.

Transfer learning involves the extraction of a meaningful latent representation from a pretrained model to use for a new, similar objective (Pan et al., 2010). We use the learned internal representations of the current state-of-art transfer component analysis (TCA) (Matasci et al., 2015) method in transfer learning to eliminate the influence of clonal proportion. TCA is able to ‘transfer’ the knowledge about one domain (called the source) to another (called the target) when given inputs of two feature matrices with different clonal proportions in the target domain and the source domain. By minimizing the distance between them and maintaining their respective data characteristics, TCA obtains dimension-reduced data in the source domain and the target domain, which makes the probability distribution of the two parts of the data more similar and shortens the data distribution distance between them, thus eliminating the difference between the different clonal proportions.

The model $f_{t}$ (target) is learned by considering the model $f_{s}$ (source), a training set that possibly follows a different data distribution. We get N samples corresponding to a certain sample purity which expressed by $D_{s}$ ,

$$D_{s}=\left\{\left(X_{s_{1}},y_{s_{1}}\right),\ldots ,\left(X_{s_{n}},y_{s_{n}}\right)\right\}$$ (1)

where $X_{s_{i}}\in \chi$ is the features, and $y_{s_{i}}$ is the binary label indicating whether it is a real variation or a false positive. The target data of other unknown sample purity is represented by $D_{t}$ , where $D_{t}=\left\{(X_{t_{1}},y_{t_{1}}\right),\ldots ,\left(X_{t_{n_{2}}},y_{t_{n_{2}}}\right)\},X_{t_{i}}\in \chi$ is the features, and $y_{t_{i}}$ is the binary label as well. We define $P\left(X_{s}\right)$ and $Q\left(X_{t}\right)$ as the marginal distributions of $X_{s}=\left\{X_{s_{i}}\right\}$ and $X_{t}=\left\{X_{t_{i}}\right\}$ , respectively. Due to different clonal proportions, *P ≠ Q* but $P\left(Y_{s}|X_{s}\right)=P\left(Y_{t}|X_{t}\right)$ . Then, our problem can be expressed as follows: by learning $D_{s}$ from the classifier, we want to predict the labels $y_{t_{i}}$ corresponding to the inputs $X_{t_{i}}$ in the target data set $D_{t}$ of unknown clonal proportion so that we can solve a learning problem in the target clonal proportion domain by utilizing the training proportion data of the source domain, correct the influence of clonal proportion, and reduce the false positive mutation calls for any clonal proportion.

Because the probability distributions of signals in the target and source domains are different, TCA assumes that a feature map can make the data distributions after mapping approximately equal. The proposed method tries to learn transfer components across domains in a reproducing kernel Hilbert space using maximum mean discrepancy (MMD), which can minimize the distribution difference in different data domains (different clonal proportions).

$$DISTANCE\left(X_{S},X_{T}\right)={\left\|\frac{n_{2}\overset{n_{1}}{\underset{i=1}{\sum }}\emptyset \left(X_{i}\right)-n_{1}\overset{n_{2}}{\underset{j=1}{\sum }}\emptyset \left(X_{j}\right)\;\;}{n_{1}n_{2}}\right\|}_{H}$$ (2)

Where $n_{1}$ represents the number of samples in the source domain, and $n_{2}$ represents the number of samples in the target domain. $\Phi \left(X_{i}\right)$ is the data distribution map of the source domain, and $\Phi \left(X_{j}\right)$ is the data distribution map of the target domain. The MMD matrix is represented as **L**, and each element $l_{i}j$ of **L** is calculated as:

$$l_{ij}=\left\{ \begin{array}{cc} \frac{1}{n_{1}^{\;2}} & \;x_{i},x_{j}\in D_{s}\\ \frac{1}{n_{2}^{\;2}} & \;x_{i},x_{j}\in D_{t}\\ -\frac{1}{n_{1}n_{2}} & otherwise \end{array}\right..$$ (3)

while the central matrix ***H*** is calculated as $H=I_{n_{1}+n_{2}}-1/\left(n_{1}+n_{2}\right)11^{T}$ . The linear kernel function $k\left(x,y\right)=x^{t}y$ is selected to construct the kernel matrix K:

$$K=\left[\begin{array}{cc} K_{s,s} & K_{s,t}\\ K_{t,s} & K_{t,t} \end{array}\right]$$ (4)

$K_{s,s}$ and $K_{t,t}$ are the Gram matrix defined on the source domain data and the target domain data in the embedded space, respectively. $K_{s,t}$ and $K_{t,s}$ are the Gram matrix defined on the cross-domain data. $K_{s,t}$ = ${K_{t,s}}^{T}$ . $1\in R^{n_{1}+n_{2}}$ is the column vector with all 1, and $I_{n_{1}+n_{2}}\in R^{\left(n_{1}+n_{2}\right)\times \left(n_{1}+n_{2}\right)}$ is the identity matrix. TCA calculates the eigen-decomposition matrix according to ${\left(KLK\;+\;\mu I\right)}^{-1}KHK$ and takes the first M eigenvectors to construct the feature data conversion matrix $W=\left\{S_{p_{j}\_p},T_{p_{j}\_p}\right\}$ from clonal proportion $p_{j}$ to purity p , where $S_{p_{j}\_p}$ is the source domain conversion matrix after dimensionality reduction, and $T_{p_{j}\_p}$ is the target domain conversion matrix after dimensionality reduction. The optimal feature dimension here M is set to 23 after multiple experiments.

### 3.2 Stage II: Extra tree classifier

False positive filtering is essentially a binary classification problem in machine learning. The normalized data set is split into training and testing sets, and the training data are passed to the model building phase for supervised analysis. In this work, the tree-like structure classifier introduces randomization in the process of constructing classifiers to creates a set of diverse classifiers and is more applicable than neural networks and linear classifiers. We use the extremely randomized trees (extra tree) algorithm as the classifier (Moosmann et al., 2008), which is a kind of integrated learning algorithm based on a parallel strategy and an average algorithm (Geurts et al., 2006).

The extra tree classifier adopts disturbance and combination technology. Compared with other classifiers, forest classifiers can use these two arrays to obtain a better fit by implementing an estimator that fits a number of randomized decision trees (a.k.a. extra trees) on variable subsamples of the dataset and uses averaging to improve the predictive accuracy and control overfitting. It not only randomly selects samples when constructing a subset of data but also extracts the features randomly (that is, when building a model, some features are used instead of all features for training). The extra tree classifier has an additional layer of randomness. When the optimal split value is selected for the continuous variable feature, a split value is randomly generated for each feature within its feature value range, and calculation is then performed to select one value for splitting. The extra tree classifier has stronger anti-noise ability and further processes the diluted sequencing signal to avoid falling into local optima or overfitting because of the tree-based combination and randomness.

### 3.3 Stage III: The Boyer-Moore majority vote algorithm

To avoid possible accidental factors in the transfer of a single source domain and to further eliminate the influence of diluted sequencing signals, we selected five low-clonal proportion samples as source domains at every 5% interval and carried out five transfers to make the model more convincing and supportive. The extra tree classifier obtained the mutations after filtering out the false positives but completed only one transfer. Each source domain may have different results. We added a majority vote algorithm—the Boyer-Moore majority vote algorithm (Cantone et al., 2003)—before the output of the algorithm to sort the final result. For each clonal proportion target domain, the output of one extra tree classifier was regarded as one vote, and the voting majority was taken as the final output.

That is, for each result transferred from a fixed sample purity $h_{i},$ (*i* = 1, 2, 3, …, a), $h_{i}^{k}\left(x\right)$ refers to the output label $y_{j}$

$$H\left(x\right)=\left\{ \begin{array}{c} y_{j}\;if\;\overset{T}{\underset{i=1}{\sum }}h_{i}^{j}\left(x\right)>0.5\overset{N}{\underset{k=1}{\sum }}\overset{T}{\underset{i=1}{\sum }}h_{i}^{k}\left(x\right)\\ reject,\;otherwise \end{array}\right.\;\left(5\right)$$

The number of source domain can be set by users. We here set it as 5 and choose sample purity in the range of 5%–30%. The formal description of the whole transfer learning approach proposed in this paper is reported in Algorithm 1.

Algorithm 1 TLsub.

**Input:** Source matrixes $D_{S_{k}}$ Target matrixes $D_{T}$

**Output**:Filtered mutation calls
1: Extract the feature datasets S

2: f**or** k = 1 to a :
3:   Construct the kernel matrix K and $X_{k},X_{p}$

4:   Build the MMD matrix **L** and central matrix **H**
5:   Calculate the eigendecomposition matrix **M**
6:   Construct the transformation matrix W

6:    **for** i = 0 to K :
7:     Build the training sample $X_{k}$

8:     Generate a base classifier
9:     **while** the node is split **do**:
10:      Select m features Randomly from M
11:      Select the optimal attribute
12:      Generate a decision tree
13:     **end**
14:     **end for**
15:    **end for**
16: Count the prediction results of all base classifiers
17: Generate the final classification result $Y_{kp}$ .
18: Obtain the result set R .
19: Vote
20: Return L

## 4 Conclusion and discussion

The proposed method deconstructs the relationship between sequencing signals and the clonal proportion by analysing signal data and introduces a transfer learning method to reconstruct a new reproducing Hilbert space, which eliminates the dilution effect of the clonal proportion on the sequencing signal and completes the filtering of false positive variation. The exact detection of subclonal mutations with diluted sequencing signals is considered fundamental for a better understanding of the mechanisms behind the expression of genes, the impact of their perturbation in the context of specific biological processes or pathways, and the wide applicability of liquid biopsy. These studies provide unprecedented opportunities for improvements in the diagnosis and treatment of different types of cancers and other human diseases. We developed an enhancement version of mutation detection by filtering false positives from next-generation sequencing data based on transfer component analysis and the Extra Tree Classifier. The proposed method can eliminate the influence of diluted sequencing signals that are mainly caused by the subclonal proportion. The main innovations are as follows: 1) the proposed method directly inputs the standard VCF file, which will not be affected by the sequencing software and detection tools. 2) The transfer learning framework was used to train on the fixed clonal proportion, which made it possible that the obtained model can be directly applied to all clonal proportions, eliminating the negative impact of diluted sequencing signals. 3) The extra tree classifier was selected to avoid overfitting and local optimization. 4) The final filtering results were obtained by the Boyer-Moore majority vote algorithm to further improve the accuracy of the algorithm. 5) Cost savings.

Given these innovations, it is encouraging to see the high accuracy of the proposed method on both simulated and real validation datasets. We carried out a series of experiments on both simulated data and real data and compared the proposed method to a set of popular variation detection tools, including Freebayes, SiNVICT, Sentieon-2019 and GATK3.8 MuTect2. The results show that the proposed method adapts well under different clonal proportions and can significantly reduce the false positive rate, and the efficiency is significantly high and stable on datasets with diluted sequencing signals. In future work, we will develop a distributed version to analyse larger datasets.

## Data Availability

The original contributions presented in the study are included in the article/Supplementary Material, further inquiries can be directed to the corresponding author.
