# Supplementary material for: TLsub: A transfer learning based enhancement to accurately detect mutations with wide-spectrum sub-clonal proportion
Source: Front Genet. 2022 Nov 22;13:981269. doi: 10.3389/fgene.2022.981269 (PMC9723383; doi:10.3389/fgene.2022.981269)
Supplement: Supplementary file 1 [file DataSheet1.docx]

Supplementary Material

**Supplementary material**

**Figures**

**
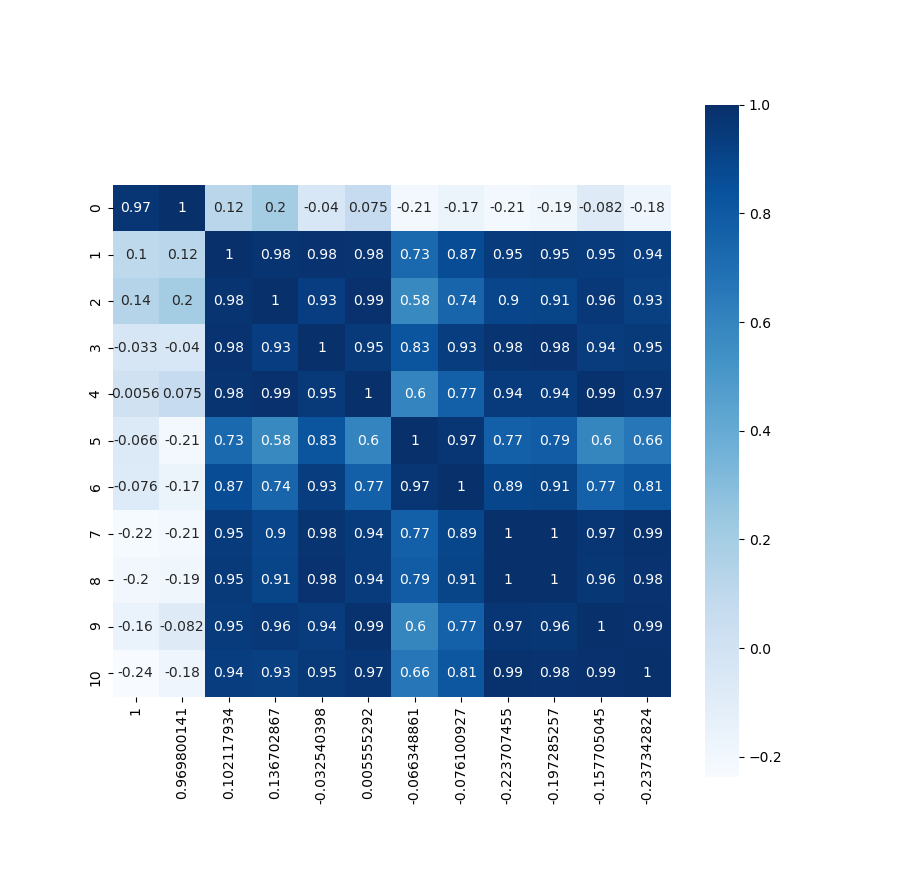
**

**
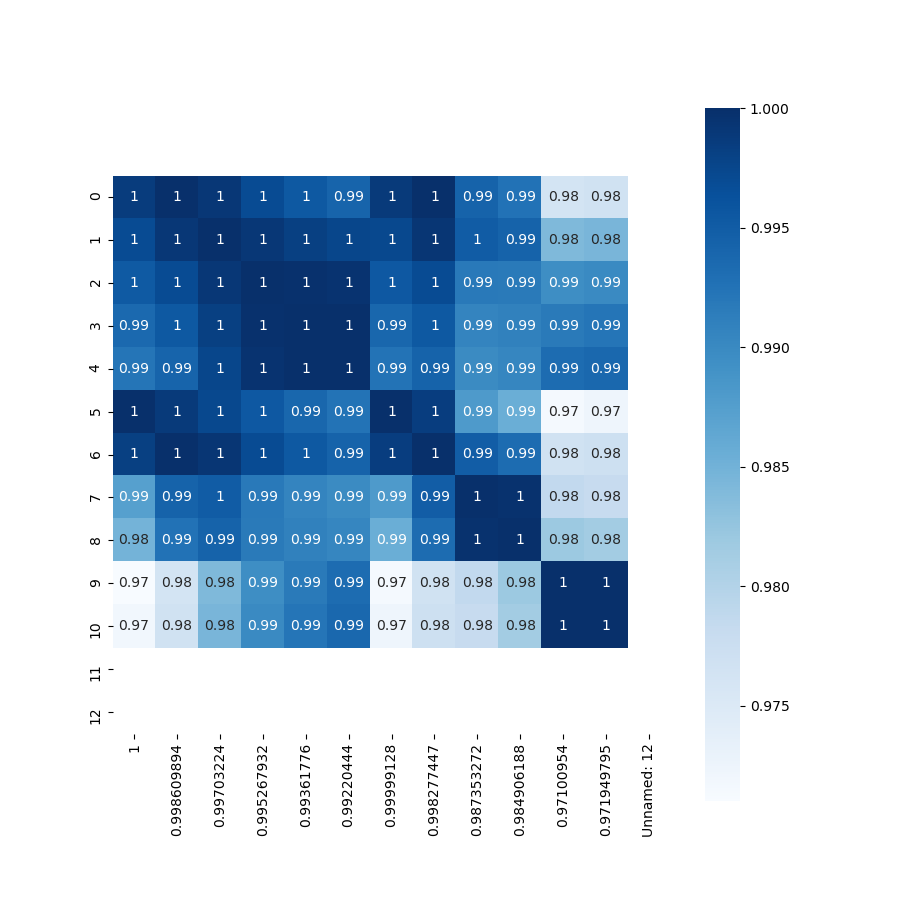
**

**
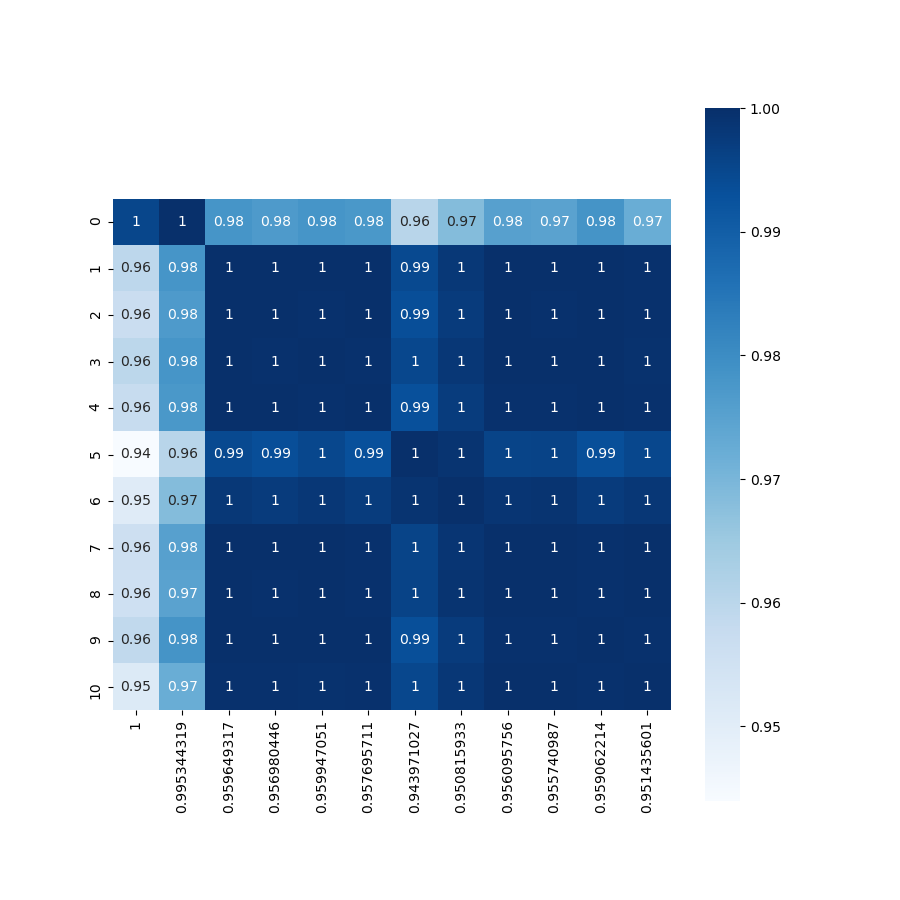
**

**
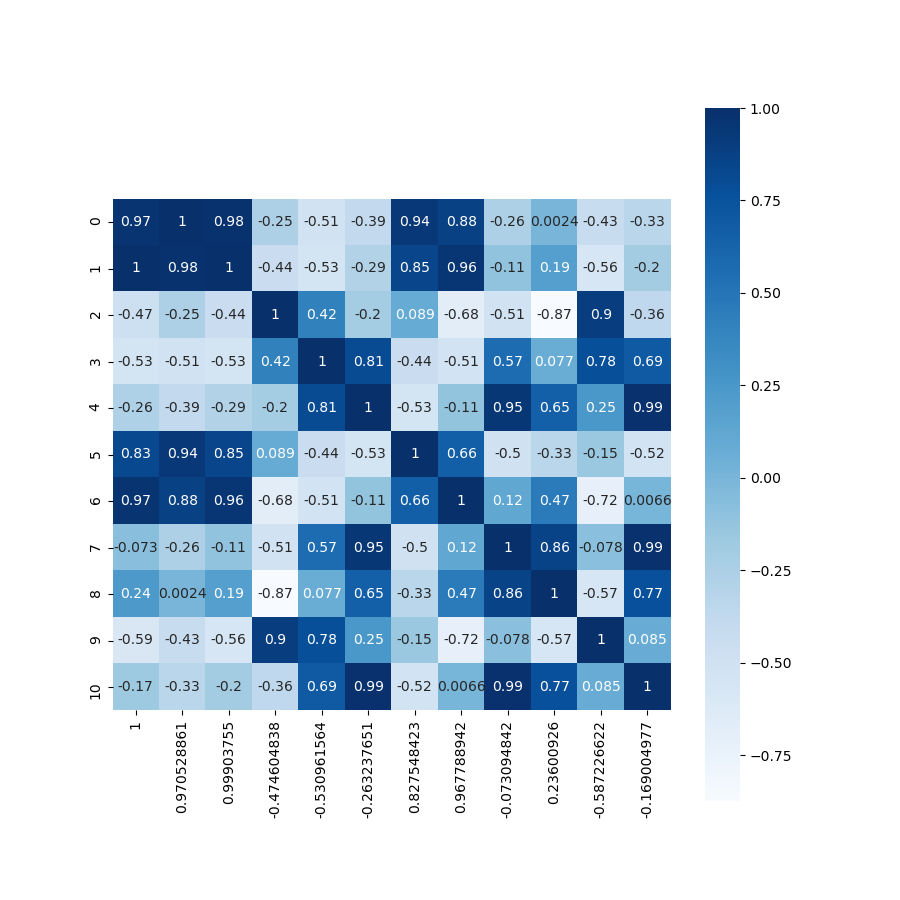
**

**
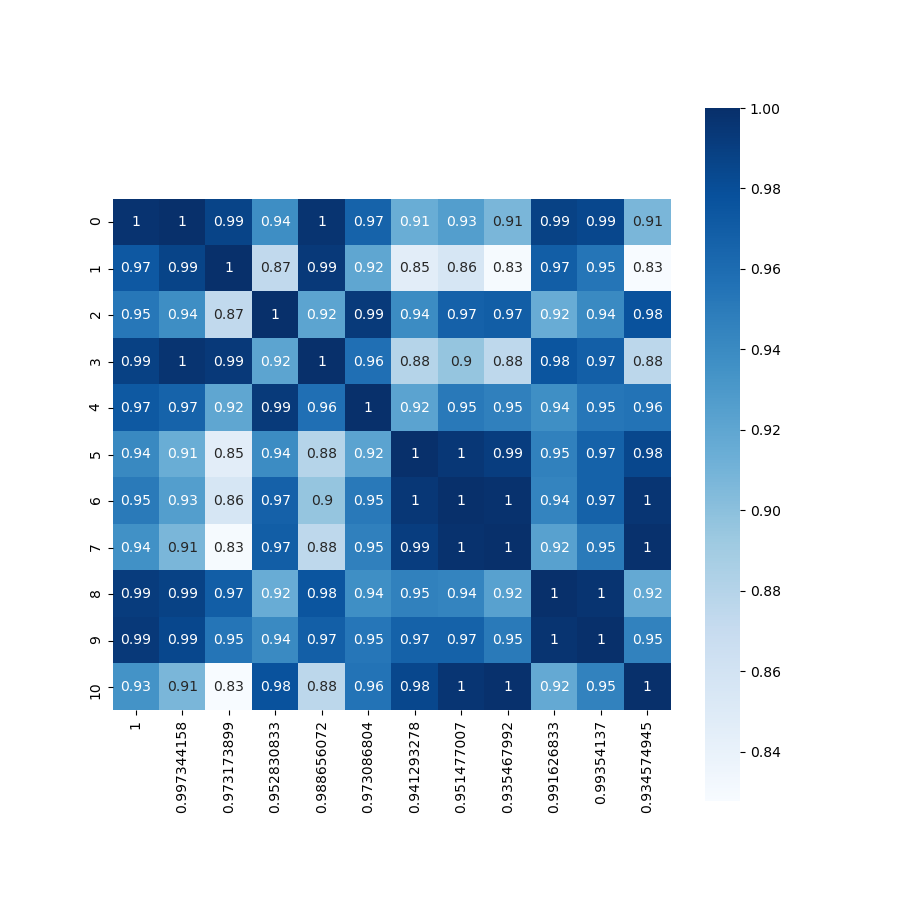
**

**
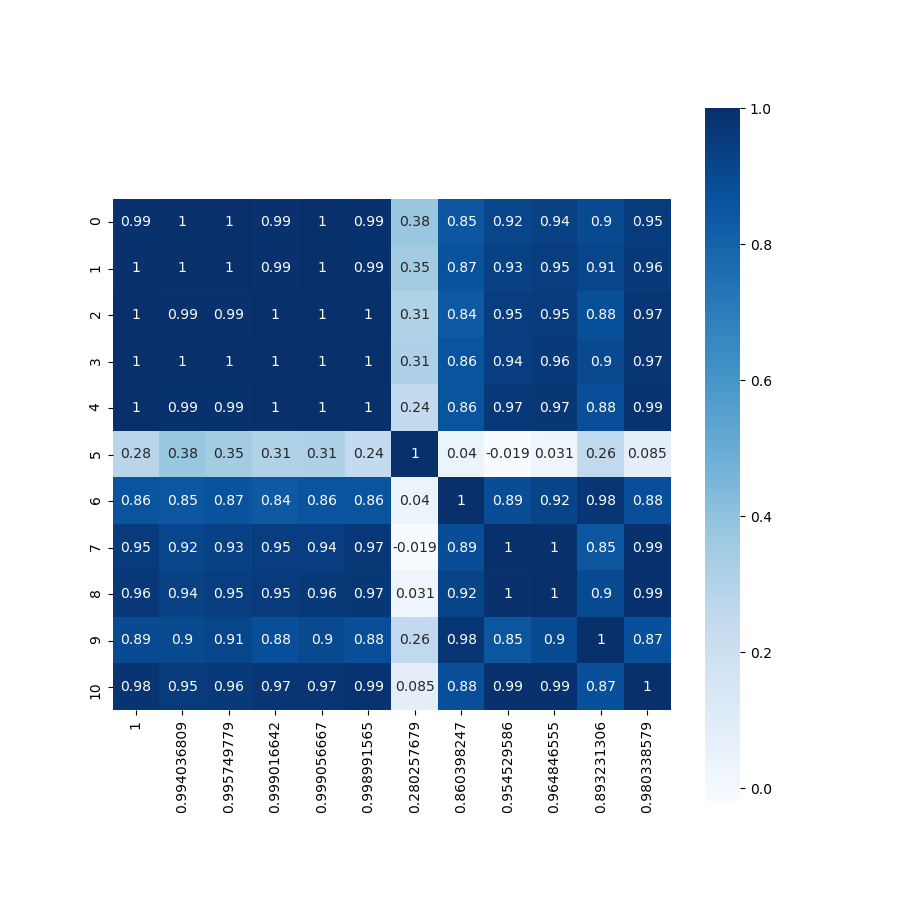
**

The correlation matrix of the six signals, CIEND, SQ, RP, CN, RO, CIPOS, between different sample purities. The heatmap figures demonstrated that the correlation ship between each signals and sample purity is different.

Tables

**Table S1. The precision of the proposed method on simulated datasets**

| Target VAF  Source VAF | Precision(%) | | | | | |
| --- | --- | --- | --- | --- | --- | --- |
|  | 0.05 | 0.10 | 0.15 | 0.20 | 0.25 | 0.30 |
| 0.05 |  | 86.13 | 87.67 | 76.41 | 84.36 | 75.69 |
| 0.10 | 87.62 |  | 88.19 | 91.32 | 87.92 | 91.14 |
| 0.15 | 85.53 | 75.48 |  | 64.19 | 89.79 | 65.62 |
| 0.20 | 84.06 | 91.23 | 85.20 |  | 88.81 | 93.39 |
| 0.25 | 82.99 | 84.88 | 89.75 | 89.82 |  | 88.32 |
| 0.30 | 81.90 | 90.60 | 88.58 | 92.56 | 91.39 |  |
| Average | 84.42 | 85.66 | 87.87 | 82.86 | 88.45 | 82.83 |
| Output | 88.10 | 90.26 | 92.25 | 92.00 | 93.21 | 94.24 |

**Table S2. The recall rate of the proposed method on simulated datasets**

| Target VAF  Source VAF | Recall (%) | | | | | |
| --- | --- | --- | --- | --- | --- | --- |
|  | 0.05 | 0.10 | 0.15 | 0.20 | 0.25 | 0.30 |
| 0.05 |  | 68.30 | 56.90 | 29.80 | 35.05 | 23.35 |
| 0.10 | 60.15 |  | 73.15 | 85.20 | 68.05 | 74.05 |
| 0.15 | 68.00 | 67.45 |  | 86.95 | 87.90 | 90.00 |
| 0.20 | 69.85 | 89.40 | 90.40 |  | 90.85 | 86.20 |
| 0.25 | 32.45 | 47.70 | 84.45 | 71.50 |  | 56.70 |
| 0.30 | 44.80 | 72.25 | 81.80 | 90.80 | 87.60 |  |
| Average | 55.05 | 69.02 | 77.34 | 72.85 | 73.89 | 66.06 |
| Output | 58.85 | 77.80 | 89.40 | 89.10 | 88.60 | 85.05 |

**Table S3. The F1-score of the proposed method on simulated datasets**

| Target VAF  Source VAF | F1-score (%) | | | | | |
| --- | --- | --- | --- | --- | --- | --- |
|  | 0.05 | 0.10 | 0.15 | 0.20 | 0.25 | 0.30 |
| 0.05 |  | 76.19 | 69.01 | 42.88 | 49.52 | 35.69 |
| 0.10 | 71.33 |  | 79.97 | 88.15 | 76.72 | 81.71 |
| 0.15 | 75.77 | 71.24 |  | 73.86 | 88.83 | 75.90 |
| 0.20 | 76.30 | 89.27 | 87.72 |  | 89.82 | 89.65 |
| 0.25 | 46.66 | 61.08 | 87.02 | 79.62 |  | 69.06 |
| 0.30 | 57.92 | 80.39 | 85.05 | 92.56 | 89.46 |  |
| Average | 65.60 | 75.63 | 81.75 | 75.41 | 78.87 | 70.40 |
| Output | 70.56 | 83.57 | 90.90 | 92.00 | 90.85 | 89.41 |

The results of parameter tuning experiments

**Table S4 The performance in terms of accuracy and AUC Score for n_estimators tuning**

| Sample purity (%) | n_estimators | 50 | 60 | 70 | 90 | 80 | 100 | 110 |
| --- | --- | --- | --- | --- | --- | --- | --- | --- |
| 5 | Accuracy | 79.65 | 80.30 | 79.98 | 79.88 | 80.05 | 79.98 | 79.85 |
|  | AUC Score | 79.65 | 80.30 | 79.98 | 79.88 | 80.05 | 79.98 | 79.85 |
| 10 | Accuracy | 87.48 | 87.30 | 87.50 | 87.68 | 87.48 | 87.63 | 87.58 |
|  | AUC Score | 87.48 | 87.30 | 87.50 | 87.68 | 87.48 | 87.63 | 87.58 |
| 15 | Accuracy | 89.80 | 89.65 | 89.88 | 89.83 | 89.90 | 89.83 | 90.00 |
|  | AUC Score | 89.80 | 89.65 | 89.88 | 89.83 | 89.90 | 89.83 | 90.00 |
| 20 | Accuracy | 91.35 | 91.33 | 91.35 | 90.85 | 91.00 | 90.78 | 90.88 |
|  | AUC Score | 91.35 | 91.33 | 91.35 | 90.85 | 91.00 | 90.78 | 90.88 |
| 25 | Accuracy | 89.90 | 90.05 | 89.88 | 89.75 | 89.88 | 89.78 | 89.90 |
|  | AUC Score | 89.90 | 90.05 | 89.88 | 89.75 | 89.88 | 89.78 | 89.90 |
| 30 | Accuracy | 89.75 | 90.00 | 90.10 | 90.30 | 90.50 | 90.23 | 90.28 |
|  | AUC Score | 89.75 | 90.00 | 90.10 | 90.30 | 90.50 | 90.23 | 90.28 |

**Table S5. The performance for max_feature tuning**

| max_features | 3 | 5 | 7 | 8 | 9 | 10 | 11 | 12 | 13 | 14 | 15 | 16 | 17 | 18 | 19 |
| --- | --- | --- | --- | --- | --- | --- | --- | --- | --- | --- | --- | --- | --- | --- | --- |
| 5 | 79.68 | 79.95 | 79.73 | 79.90 | 80.15 | 79.80 | 80.30 | 80.40 | 80.23 | 80.70 | 80.90 | 81.23 | 81.33 | 80.65 | 81.25 |
| 10 | 87.38 | 87.93 | 87.58 | 87.63 | 87.68 | 87.50 | 87.65 | 87.78 | 87.48 | 87.73 | 87.88 | 88.18 | 87.50 | 87.88 | 87.45 |
| 15 | 89.23 | 89.23 | 89.50 | 89.68 | 89.73 | 89.50 | 89.93 | 89.60 | 89.53 | 90.00 | 89.68 | 89.60 | 89.60 | 89.93 | 89.25 |
| 20 | 90.73 | 90.93 | 90.95 | 91.43 | 91.13 | 91.78 | 91.73 | 91.18 | 91.53 | 91.20 | 91.73 | 91.30 | 91.35 | 91.73 | 91.58 |
| 25 | 89.58 | 89.88 | 90.15 | 90.40 | 90.80 | 90.70 | 90.58 | 91.18 | 90.93 | 90.78 | 91.18 | 91.25 | 90.95 | 91.08 | 91.30 |
| 30 | 89.33 | 90.58 | 90.25 | 91.48 | 91.30 | 90.98 | 91.33 | 91.28 | 91.15 | 91.90 | 91.35 | 91.78 | 91.03 | 91.40 | 92.05 |

**Table S6. The performance for max_depth tuning**

| max_depth | 13 | 15 | 17 | 19 | 21 | 23 | 25 | 27 | 29 | 31 | 33 | 35 | 37 | 39 | 40 | 41 | 42 | 43 | 44 | 45 | 47 | 49 |
| --- | --- | --- | --- | --- | --- | --- | --- | --- | --- | --- | --- | --- | --- | --- | --- | --- | --- | --- | --- | --- | --- | --- |
| 5 | 79.38 | 80.00 | 79.45 | 80.13 | 79.50 | 79.75 | 79.83 | 79.80 | 80.30 | 79.88 | 79.88 | 80.08 | 80.05 | 80.10 | 80.13 | 80.03 | 80.08 | 80.05 | 80.05 | 80.05 | 80.05 | 80.05 |
| 10 | 87.53 | 87.20 | 87.35 | 87.73 | 87.70 | 87.55 | 87.75 | 87.58 | 87.38 | 87.43 | 87.60 | 87.38 | 87.48 | 87.43 | 87.53 | 87.58 | 87.48 | 87.48 | 87.48 | 87.48 | 87.48 | 87.48 |
| 15 | 85.85 | 87.43 | 88.83 | 89.23 | 89.25 | 89.78 | 89.55 | 89.73 | 89.50 | 89.95 | 90.00 | 89.83 | 89.88 | 89.83 | 89.85 | 89.93 | 89.90 | 89.90 | 89.90 | 89.90 | 89.90 | 89.90 |
| 20 | 89.55 | 89.85 | 90.23 | 91.23 | 91.08 | 91.05 | 91.08 | 91.80 | 91.00 | 91.05 | 91.40 | 91.18 | 90.98 | 90.95 | 90.90 | 90.98 | 90.98 | 90.98 | 91.00 | 90.98 | 91.00 | 91.00 |
| 25 | 85.63 | 88.33 | 88.80 | 89.40 | 89.75 | 89.58 | 89.60 | 89.60 | 90.10 | 89.95 | 89.90 | 89.95 | 89.88 | 89.93 | 89.80 | 89.80 | 89.85 | 89.88 | 89.88 | 89.88 | 89.88 | 89.88 |
| 30 | 84.93 | 87.10 | 88.30 | 89.30 | 90.00 | 89.85 | 90.63 | 90.45 | 90.40 | 90.10 | 90.43 | 90.38 | 90.38 | 90.40 | 90.45 | 90.53 | 90.50 | 90.50 | 90.50 | 90.50 | 90.50 | 90.50 |

**Table S7 The performance for min_samples_split tuning**

| min_samples_split | 50 | 70 | 90 | 110 | 130 | 150 | 170 | 190 |
| --- | --- | --- | --- | --- | --- | --- | --- | --- |
| max_depth | 3 | | | | | | | |
| 5 | 75.20% | 75.38 | 75.50 | 75.55 | 75.20 | 75.23 | 75.33 | 75.00 |
| 10 | 80.48 | 80.70 | 80.90 | 80.68 | 80.88 | 80.45 | 80.73 | 80.50 |
| 15 | 74.00 | 74.13 | 73.80 | 74.05 | 74.15 | 73.80 | 73.68 | 73.78 |
| 20 | 76.63 | 77.23 | 76.85 | 77.18 | 77.20 | 77.18 | 76.58 | 76.73 |
| 25 | 74.60 | 74.53 | 74.48 | 74.63 | 74.70 | 74.83 | 74.68 | 74.73 |
| 30 | 76.58 | 76.50 | 76.15 | 76.18 | 76.08 | 75.95 | 75.85 | 75.60 |
| max_depth | 5 | | | | | | | |
| 5 | 77.95 | 77.75 | 77.60 | 77.35 | 77.05 | 77.43 | 77.30 | 76.95 |
| 10 | 84.73 | 84.53 | 84.03 | 83.80 | 83.38 | 83.48 | 83.53 | 83.58 |
| 15 | 76.93 | 77.28 | 76.58 | 76.20 | 76.23 | 76.33 | 75.85 | 75.90 |
| 20 | 80.93 | 81.33 | 79.83 | 79.53 | 79.73 | 78.80 | 78.95 | 79.25 |
| 25 | 78.23 | 78.55 | 78.13 | 77.35 | 76.88 | 77.00 | 77.03 | 77.30 |
| 30 | 77.70 | 77.25 | 77.18 | 77.75 | 77.55 | 78.68 | 78.03 | 77.28 |
| min_samples_split | 7 | | | | | | | |
| 5 | 78.35 | 77.55 | 77.75 | 77.48 | 77.45 | 77.05 | 77.18 | 77.43 |
| 10 | 87.00 | 86.35 | 86.18 | 86.38 | 85.85 | 85.73 | 85.70 | 85.25 |
| 15 | 78.88 | 79.08 | 78.68 | 79.25 | 78.95 | 78.60 | 78.50 | 78.95 |
| 20 | 83.78 | 83.75 | 83.70 | 83.48 | 82.53 | 82.00 | 82.90 | 83.20 |
| 25 | 79.38 | 78.88 | 79.73 | 80.03 | 79.50 | 79.20 | 79.35 | 79.38 |
| 30 | 81.03 | 78.45 | 81.10 | 80.53 | 80.58 | 78.88 | 79.25 | 79.73 |
| max_depth | 9 | | | | | | | |
| 5 | 78.83 | 78.10 | 77.45 | 77.28 | 77.15 | 76.25 | 76.00 | 75.70 |
| 10 | 86.18 | 86.20 | 86.63 | 85.53 | 85.83 | 85.80 | 85.58 | 86.23 |
| 15 | 81.25 | 80.93 | 80.78 | 80.38 | 79.85 | 80.48 | 80.60 | 81.40 |
| 20 | 85.73 | 86.50 | 85.95 | 85.10 | 85.03 | 85.25 | 84.80 | 84.45 |
| 25 | 81.98 | 81.58 | 80.78 | 81.05 | 80.93 | 80.38 | 80.35 | 79.98 |
| 30 | 81.13 | 80.73 | 80.73 | 78.40 | 80.05 | 81.00 | 79.93 | 80.08 |
| max_depth | 11 | | | | | | | |
| 5 | 78.33 | 76.43 | 76.78 | 76.88 | 75.83 | 76.23 | 76.40 | 76.63 |
| 10 | 86.35 | 86.10 | 87.03 | 86.55 | 85.20 | 86.13 | 86.45 | 86.08 |
| 15 | 82.75 | 80.85 | 80.68 | 80.60 | 82.33 | 81.95 | 81.80 | 81.60 |
| 20 | 87.73 | 86.78 | 86.33 | 85.28 | 86.40 | 86.85 | 86.28 | 86.30 |
| 25 | 83.00 | 82.43 | 81.58 | 81.93 | 81.28 | 81.53 | 81.00 | 81.08 |
| 30 | 82.83 | 82.88 | 81.78 | 81.30 | 81.08 | 80.63 | 79.88 | 79.73 |
| max_depth | 13 | | | | | | | |
| 5 | 76.98 | 76.35 | 76.28 | 76.93 | 76.53 | 76.13 | 75.85 | 75.38 |
| 10 | 86.73 | 86.75 | 86.05 | 86.98 | 86.63 | 86.50 | 86.78 | 86.95 |
| 15 | 83.25 | 82.88 | 82.63 | 83.28 | 83.63 | 82.90 | 82.13 | 81.75 |
| 20 | 88.08 | 87.18 | 87.63 | 86.38 | 87.15 | 87.30 | 86.78 | 87.18 |
| 25 | 84.60 | 82.88 | 83.23 | 83.50 | 82.60 | 82.38 | 81.83 | 81.18 |
| 30 | 83.10 | 82.73 | 80.58 | 82.08 | 81.38 | 79.10 | 80.43 | 80.38 |

**Table S8. The performance for min_samples_leaf tuning**

| min_samples_leaf | 1 | 2 | 3 | 4 | 5 | 6 | 7 | 8 | 9 | 10 |
| --- | --- | --- | --- | --- | --- | --- | --- | --- | --- | --- |
| 5 | **80.05** | 79.63 | 78.08 | 77.80 | 77.73 | 76.88 | 76.30 | 76.35 | 75.48 | 76.35 |
| 10 | 87.48 | **87.63** | 87.73 | 87.75 | 86.90 | 87.60 | 86.35 | 87.68 | 86.58 | 86.23 |
| 15 | **89.90** | 88.98 | 88.33 | 87.23 | 87.08 | 86.20 | 86.08 | 84.68 | 84.90 | 84.68 |
| 20 | **91.00** | 90.55 | 90.23 | 89.75 | 89.33 | 89.48 | 88.45 | 88.98 | 88.13 | 87.55 |
| 25 | **89.88** | 89.10 | 87.98 | 87.95 | 86.50 | 85.75 | 85.25 | 84.65 | 83.85 | 83.60 |
| 30 | **90.50** | 89.48 | 87.73 | 87.45 | 87.08 | 85.98 | 85.43 | 83.63 | 84.15 | 82.73 |

The best results are highlighted in boldface

**Table S9. The performance results of the proposed method varying the minimum applicable number of samples**

| Purity | proportion of positive cases | 10% | 20% | 30% |
| --- | --- | --- | --- | --- |
| 5 | Accuracy | 79.90% | 76.95% | 78.95% |
|  | recall_score | 70.35% | 62.00% | 73.90% |
|  | f1_score | 77.78% | 72.90% | 77.83% |
|  | precision_score | 86.96% | 88.45% | 82.20% |
|  | roc_auc_score | 79.90% | 76.95% | 78.95% |
| 10 | Accuracy | 87.90% | 88.68% | 83.63% |
|  | recall_score | 84.80% | 88.70% | 80.50% |
|  | f1_score | 87.51% | 88.68% | 83.10% |
|  | precision_score | 90.41% | 88.66% | 85.87% |
|  | roc_auc_score | 87.90% | 88.68% | 83.63% |
| 15 | Accuracy | 89.58% | 85.70% | 83.25% |
|  | recall_score | 92.15% | 90.30% | 83.80% |
|  | f1_score | 89.84% | 86.33% | 83.34% |
|  | precision_score | 87.64% | 82.69% | 82.89% |
|  | roc_auc_score | 89.58% | 85.70% | 83.25% |
| 20 | Accuracy | 91.63% | 90.65% | 86.88% |
|  | recall_score | 91.95% | 91.35% | 85.55% |
|  | f1_score | 91.65% | 90.72% | 86.70% |
|  | precision_score | 91.36% | 90.09% | 87.88% |
|  | roc_auc_score | 91.63% | 90.65% | 86.88% |
| 25 | Accuracy | 90.70% | 89.93% | 85.13% |
|  | recall_score | 90.10% | 89.25% | 80.55% |
|  | f1_score | 90.64% | 89.86% | 84.41% |
|  | precision_score | 91.19% | 90.47% | 88.66% |
|  | roc_auc_score | 90.70% | 89.93% | 85.13% |
| 30 | Accuracy | 90.03% | 85.55% | 84.85% |
|  | recall_score | 88.35% | 80.35% | 83.05% |
|  | f1_score | 89.86% | 84.76% | 84.57% |
|  | precision_score | 91.41% | 89.68% | 86.15% |
|  | roc_auc_score | 90.03% | 85.55% | 84.85% |

**Table S10. The performance results of the proposed method varying the range of label error rate**

| Purity | proportion of positive cases | 10% | 20% | 30% |
| --- | --- | --- | --- | --- |
| 5 | Accuracy | 79.90% | 76.95% | 78.95% |
|  | recall_score | 70.35% | 62.00% | 73.90% |
|  | f1_score | 77.78% | 72.90% | 77.83% |
|  | precision_score | 86.96% | 88.45% | 82.20% |
|  | roc_auc_score | 79.90% | 76.95% | 78.95% |
| 10 | Accuracy | 87.90% | 88.68% | 83.63% |
|  | recall_score | 84.80% | 88.70% | 80.50% |
|  | f1_score | 87.51% | 88.68% | 83.10% |
|  | precision_score | 90.41% | 88.66% | 85.87% |
|  | roc_auc_score | 87.90% | 88.68% | 83.63% |
| 15 | Accuracy | 89.58% | 85.70% | 83.25% |
|  | recall_score | 92.15% | 90.30% | 83.80% |
|  | f1_score | 89.84% | 86.33% | 83.34% |
|  | precision_score | 87.64% | 82.69% | 82.89% |
|  | roc_auc_score | 89.58% | 85.70% | 83.25% |
| 20 | Accuracy | 91.63% | 90.65% | 86.88% |
|  | recall_score | 91.95% | 91.35% | 85.55% |
|  | f1_score | 91.65% | 90.72% | 86.70% |
|  | precision_score | 91.36% | 90.09% | 87.88% |
|  | roc_auc_score | 91.63% | 90.65% | 86.88% |
| 25 | Accuracy | 90.70% | 89.93% | 85.13% |
|  | recall_score | 90.10% | 89.25% | 80.55% |
|  | f1_score | 90.64% | 89.86% | 84.41% |
|  | precision_score | 91.19% | 90.47% | 88.66% |
|  | roc_auc_score | 90.70% | 89.93% | 85.13% |
| 30 | Accuracy | 90.03% | 85.55% | 84.85% |
|  | recall_score | 88.35% | 80.35% | 83.05% |
|  | f1_score | 89.86% | 84.76% | 84.57% |
|  | precision_score | 91.41% | 89.68% | 86.15% |
|  | roc_auc_score | 90.03% | 85.55% | 84.85% |
